# Effects of histone methylation modification on low temperature seed germination and growth of maize

**DOI:** 10.1038/s41598-023-32451-5

**Published:** 2023-03-30

**Authors:** Xin Qi, Chang Wan, Xing Zhang, Weifeng Sun, Rui Liu, Zhennan Wang, Zhenhui Wang, Fenglou Ling

**Affiliations:** 1 Faculty of Agronomy, Jilin Agricultural University, Changchun, Jilin China; 2 Institute of Grassland and Ecology, Jilin Academy of Agricultural Sciences, Changchun, Jilin China

**Keywords:** Molecular biology, Plant sciences

## Abstract

Low temperature is a limiting factor of seed germination and plant growth. Although there is a lot information on the response of maize to low temperatures, there is still poorly description of how histone methylation affects maize germination and growth development at low temperatures. In this study, the germination rate and physiological indexes of wild-type maize inbred lines B73 (WT), SDG102 silencing lines (AS), SDG102 overexpressed lines (OE) at germination stage and seedling stage were measured under low temperature stress (4 ℃), and transcriptome sequencing was applied to analyze the differences of gene expression in panicle leaves among different materials. The results showed that the germination rate of WT and OE maize seeds at 4 ℃ was significantly lower than 25 ℃. The content of MDA, SOD and POD of 4 ℃ seeding leaves higher than contrast. Transcriptome sequencing results showed that there were 409 different expression genes (DEGs) between WT and AS, and the DEGs were mainly up-regulated expression in starch and sucrose metabolism and phenylpropanoid biosynthesis. There were 887 DEGs between WT and OE, which were mainly up-regulated in the pathways of plant hormone signal transduction, porphyrin and chlorophyll metabolism. This result could provide a theoretical basis for analyzing the growth and development of maize from the perspective of histone methylation modification.

## Introduction

Low temperature is an important limiting factor in plant growth, photosynthesis, uptake of water and nutrients, as well as plant productivity^1^. Extreme temperatures give rise to an oxidative damage in plants, resulting in excessive accumulation of Reactive Oxygen Species (ROS) produced as a result of oxidative damage. To alleviate oxidative damage, plants have evolved protective enzymatic and non-enzymatic defense systems to detoxify ROS and reduce oxidative stress^2^. Temperature is one of the most critical factors affecting the germination of seeds^3^. The ability of seeds to germinate and seedlings to establish determines to some extent the later growth and development of the plant. Maize is one of the three major grain crops in China. It is an important food material and also used in industry and medical treatment^4^. Among them, DNA methylation is an important epigenetic marker, under the catalysis of DNA methyltransferase, the specific bases on the DNA sequence can be inherited to the new offspring DNA with the DNA replication process^5^. Histone modification refers to the process of methylation, acetylation, phosphorylation, adenylation, ubiquitination, and ADP ribosylation of histones under the action of related^6^. Epigenetic refers to the absence of changes in an organism's DNA sequence, resulting in heritable changes in gene expression or cell phenotypes. The main modes of epigenetic type include DNA methylation, histone modification, chromosome remodeling and non-coding RNA, etc.^7^. Histone methylation is an important part of histone modification, which regulates the expression level of related genes through different sites and different degrees of methylation, and affects the growth and development of plants^8^. Numerous studies have shown that H3 and H4 histone modifications are relevant with the expression of certain genes related to plant development, senescence, flowering and stress response^9–12^. SDG (SET Domain Group) gene family encoding histone methyltransferase can participate in histone methylation. It has the function of regulating seed germination, stress response and other biological processes^13,14^. The SDG gene family is considered to be a class of proteins with conserved SET domains, only SDG proteins can act as histone lysine methyltransferases (HKMTases) to play an important role in plant growth and development^15^. Histone methylation can occur to varying degrees (monomethylation, dimethylation and trimethylation) and on different lysine residues (e.g., H_3_K_4_, H_3_K_9_, H_3_K_27_, and H_3_K_36_)^16^, Histone H_3_K_9_ and H_3_K_27_ methylation is often associated with transcriptional gene silencing, while histone H_3_K_4_ and H_3_K_36_ are associated with gene activation^17^. So far, more than 30 kinds of histone lysine methylases have been re-ported in Arabidopsis, and these 30 kinds are divided into 5 categories, first category: E(Z) family, Drosophila PcG (Polycomd Group) proteins with conserved SET domains, which function as H_3_K_27_ methyltransferases. The second category: the ASH1 family, which catalyzes the methylation of H_3_K_36_. The third category: trithorax family, TrxG proteins mainly maintain the activation state of transcription. The fourth category: proteins containing SET and PHD domains, there are few related reports at present. The fifth category: SU (VAR) family, similar to PcG proteins to maintain transcriptional re-pression functions^18^. Plant genome analysis of the SDG gene family was performed in Arabidopsis, maize and rice, and functional studies showed that SDG genes are in-volved in the control of plant development^19^, the gene SDG26 in Arabidopsis thaliana and the gene SDG708 in rice are both homologous to the SDG102 gene in maize, have H_3_K_36_ methyltransferase activity, and participate in histone methylation modification. SDG26 and SDG708 have important roles in regulating plant transcription, growth and development^17^.

SUVH5 as an H_3_K_9_ methyltransferase, restrains the expression of key genes related to seed germination, such as ABA biosynthesis and signal transduction^20^. PIF1 (Phytochrome Interacting Factor 1) which inhibits light-dependent seed germination, and mutations in the short-day early flowering (EFS) gene encoding H_3_K_4_ and H_3_K_36_ methyltransferases, the levels of H_3_K_36_me2 and H3K36me3 at the PIF1 locus, and reduced PIF1 mRNA expression in assimilated seeds^21^. SDG711 is involved in starch accumulation and thus controls normal seed^22^. The down-regulation of SDG725 gene in rice leads to extensive defects, such as dwarfing and internode shortening, etc. In SDG725 silenced lines, H_3_K_36_me2/3 levels were reduced in certain regions of brassinosteroid-associated^23^. Overexpression of SDG701 promoted rice flowering, while silencing of SDG701 delayed rice^24^.

Transcriptome sequencing (RNA-Seq) uses high-throughput sequencing technology to sequence all RNA reverse transcribed to cDNA libraries in tissues or cells. Transcriptome sequencing was used to analyze natural senescence and premature senescence of tobacco, and to find differences in enrichment pathways, important gene functions and signal transduction, in order to explain the key factors regulating senescence^25^. Transcriptome sequencing technology was used to study the ear leaves blade of premature senescence maize leaves at the late senescence stage, analyze the different expression genes and metabolic pathways, and show the metabolic regulatory network related to maize leaf senescence^26^. At present, about histone methylation studies are mainly focused on monocotyledons rice^27^ and dicotyledons Arabidopsis^9^, but there are few reports on epigenetic analysis of maize leaf growth and development.

In this study, wild-type maize inbred lines B73, SDG102 silencing lines and SDG102 overexpressed lines were used as materials. The germination rate of seeds at germination stage under low temperature stress was determined. Physiological and biochemical indexes of seedling leaves under low temperature stress were measured. The phenotype of maize leaves, plant height, number of maize leaves and chlorophyll content of maize were measured in the field. We used transcriptomic data to expressed genes and analyze the effects of histone methylation modification on seed germination and seedling physiological on low temperature. Our results may provide a theoretical basis for the analysis of the effects of low temperature on maize germination and growth from the perspective of histone methylation modifications.

## Results

### Influence of low temperature on maize seed germination

**Figure 1:**
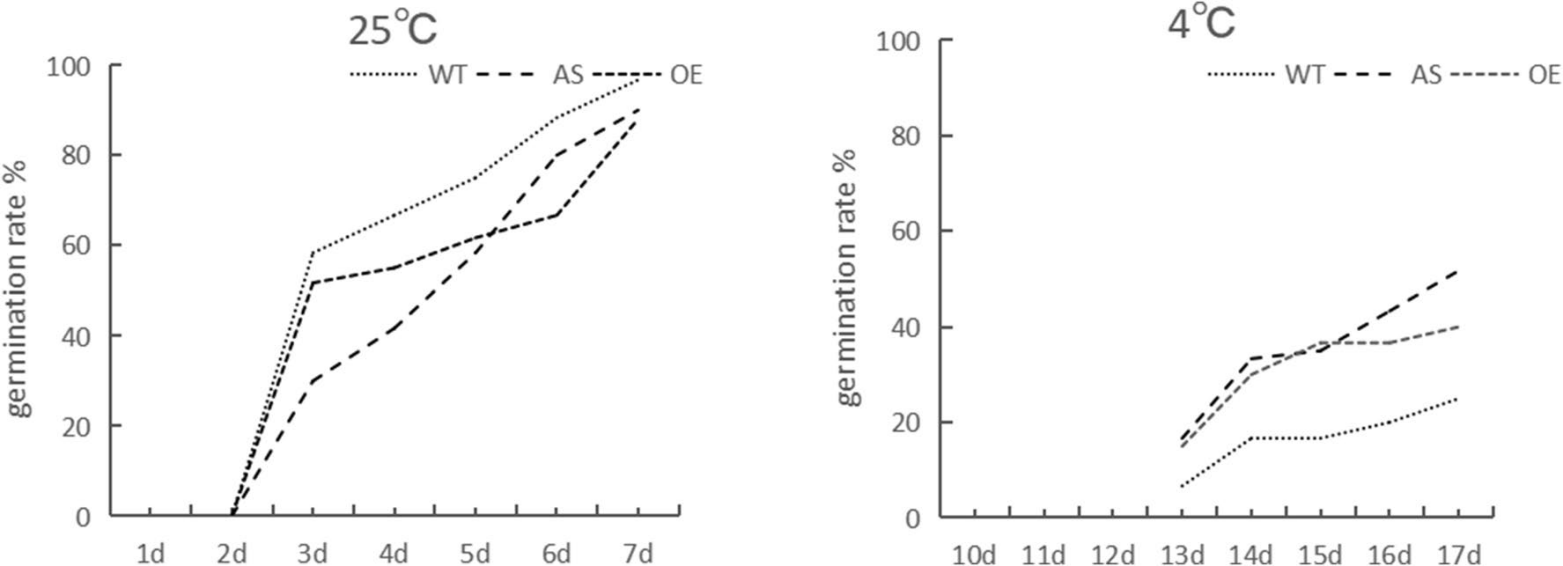
The trend of germination rate with germinating days.

Low temperature is an important factor to inhibit seed germination. The germination rate of maize seeds at 4 ℃ was lower than the control, and WT and OE were significant differences before and after low temperature stress, but AS showed no significant differences (Table 1). The number of seed germination increased with the extension of treatment days, the germination days of seeds under low temperature stress were about 11 days later than that of normal temperature control (Fig. 1).

**Table 1:** Effects of different temperature on seed germination characteristics of maize.

Material	Germination rate (%)	
	25 ℃	4 ℃
WT	96.7	25*
AS	90	51.7*
OE	88.3	40*

Note: compared with normal temperature control, "*" indicate by the t-test analysis, P < 0.05.

### Effects of low temperature on physiological and biochemical indexes of maize seedling leaves

**Figure 2:**
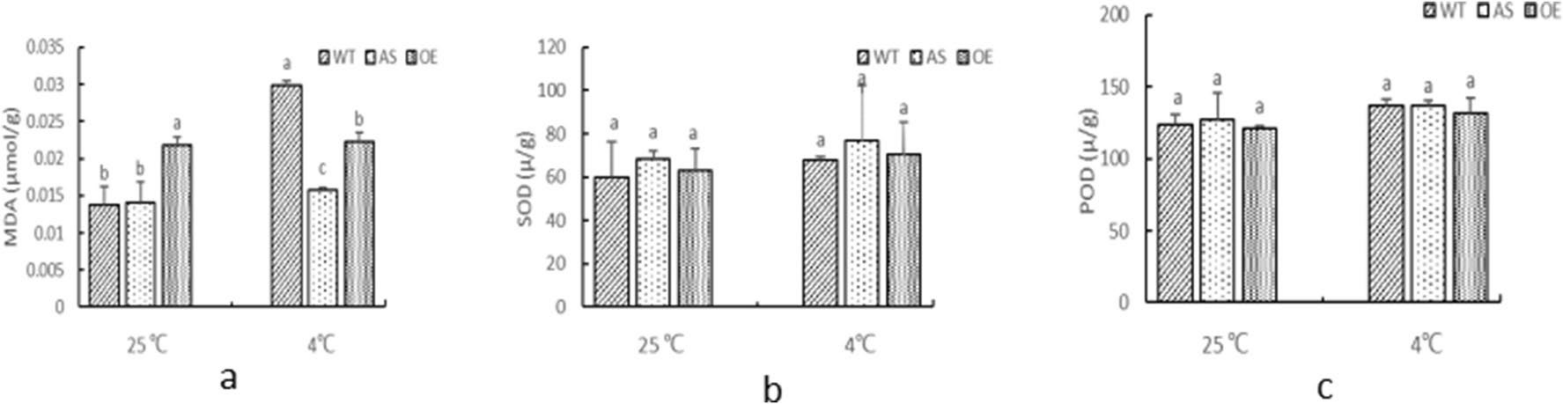
Effects of low temperature on physiological indexes of maize seedling leaves. (**a**) Effects of low temperature on MDA content in maize seedling leaves; (**b**) effects of low temperature on SOD enzyme activity in maize seedling leaves; (**c**) effects of low temperature on POD activity in maize seedling leaves. Different lowercase letters indicate significant differences in physiological indicators between plants (*P* < 0.05).

Compared with the 25 ℃, MDA, SOD and POD of all materials under 4 ℃ showed an increasing trend. MDA of WT showed significant difference before and after low temperature stress, while MDA of AS and OE showed no significant difference before and after low temperature stress. MDA of AS and OE under 4 ℃ showed significant difference compared with WT (Fig. 2a). SOD of WT, AS and OE showed no significant difference before and after low temperature stress, and SOD of AS and OE under low temperature stress showed no significant difference compared with WT (Fig. 2b). There was no significant difference in POD of WT, AS and OE before and after low temperature stress, and no significant difference in POD of AS and OE under low temperature stress compared with WT (Fig. 2c). It indicated that low temperature stress had little effect on the transgenic SDG102 material.

### Analysis of plant agronomic characters and chlorophyll content in leaves

Height and number of fully expanded leaves of 40 WT, AS and OE plants were measured at maturity. Compared with WT, the plant height of AS increased by about 18 cm, while that of OE decreased by about 24 cm. The plant height of AS and OE was significantly different from that of the control. The number of AS and OE leaves was more than that in the control group, and the difference was not significant. Compared with WT, the date of AS powder was delayed by about 4 days, reaching an extremely significant difference level, while the date of OE powder was advanced by about 2 days, showing no significant difference (Table 2).

**Table 2:** Agronomic traits of maize plants.

Material	Plant height (cm)	Leaf number	Powder days
WT	201.86 ± 14.4	21.9 ± 0.6	78 ± 3.52
AS	219.61 ± 10.09**	22.4 ± 0.8	82.33 ± 1.6**
OE	177.44 ± 14.64**	22.7 ± 0.8	76.99 ± 2.67

Note: indicate by the t-test analysis, ‘*’: P < 0.05; ‘**’: P < 0.01.

**Figure 3:**
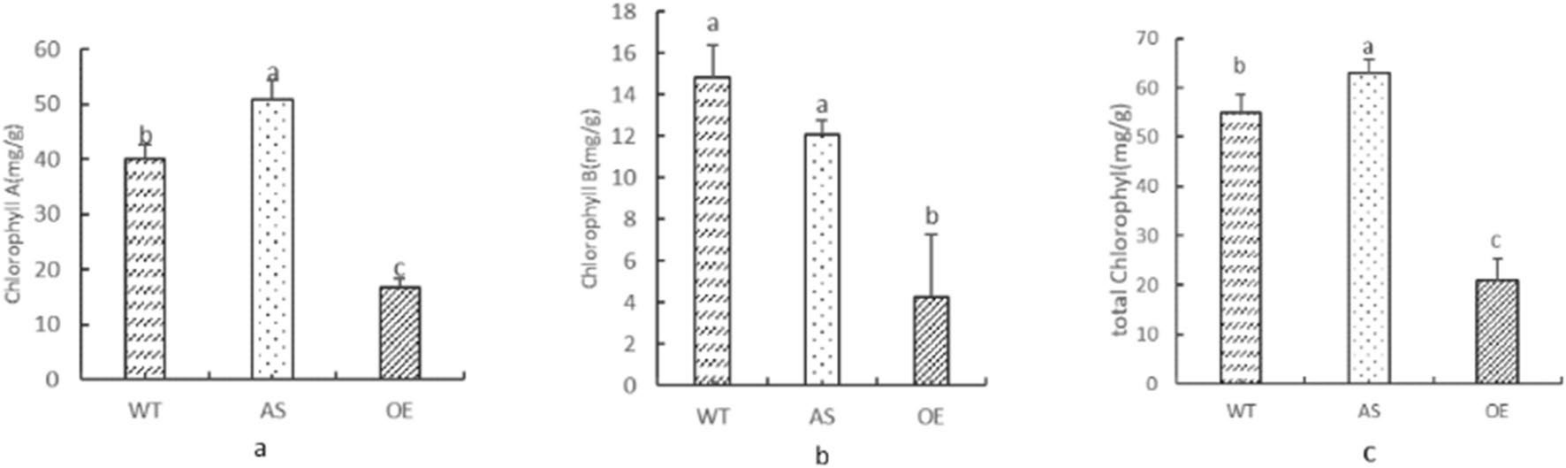
The content of Chlorophyll A, Chlorophyll B, total Chlorophyll content in different materials. (**a**) The content of Chlorophyll A; (**b**) the content of Chlorophyll B; (**c**) the content of total chlorophyll. Different lowercase letters indicate significant differences in Chlorophyll A, Chlorophyll B, total Chlorophyll content between materials (*P* < 0.05).

With the prolongation of pollination days, there were obvious phenotypic differences in the leaves at ear position of the three materials. The leaves at ear position after pollination for 30 days were selected to measure the chlorophyll content. AS chlorophyll A content and total chlorophyll content were higher than WT and OE, while OE was lower than WT and AS in chlorophyll A, chlorophyll B and total chlorophyll content. There were significant differences in chlorophyll A and total chlorophyll content be-tween AS and WT, and significant differences in chlorophyll A, chlorophyll B and total chlorophyll content between OE and WT (Fig. 3). These results suggested that the difference of chlorophyll content in maize leaves at ear position may be caused by his-tone methylation during late pollination in field.

### Quality analysis of transcriptome sequencing

The mapping of Reads between samples and reference genomes ranged from 80.26 to 94.93%, resulting in 52.33 Gb of Clean Data, with the percentage of Q30 base of each sample not less than 94.82% (Table 3). The sequencing Data had high reliability and could be used for subsequent bioinformatics analysis.

**Table 3:** Statistical table of sequencing data.

Sample	Clean reads	Clean bases	Mapped reads	% ≥ Q30
WT1	21,150,178	6,331,758,956	34,456,171 (81.46%)	94.82%
WT2	22,991,413	6,876,109,798	43,649,464 (94.93%)	95.76%
WT3	19,417,182	5,812,057,660	35,295,721 (90.89%)	95.14%
AS1	17,206,933	5,150,862,508	28,044,456 (81.49%)	95.38%
AS2	18,932,766	5,662,683,094	33,455,901 (88.35%)	95.99%
AS3	18,867,288	5,642,301,522	30,287,186 (80.26%)	95.15%
OE1	19,035,256	5,692,305,426	32,389,468 (85.08%)	95.83%
OE2	18,618,317	5,562,529,118	31,890,600 (85.64%)	95.98%
OE3	18,741,785	5,604,250,638	30,808,961 (82.19%)	95.97%

### Analysis of DEGs

**Figure 4:**
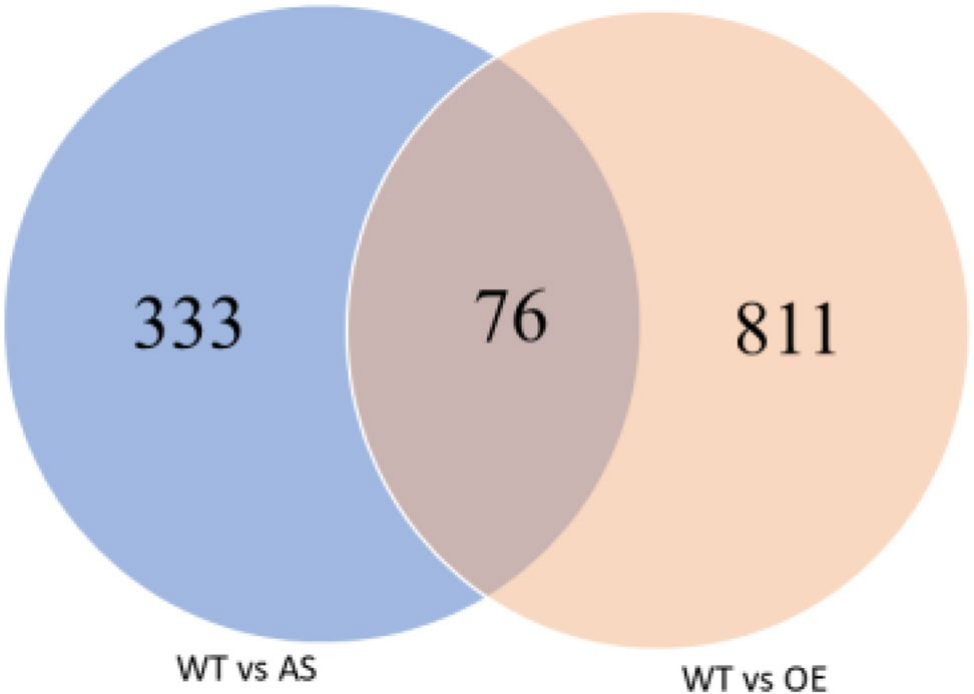
Venn diagram of different expression genes.

There were 409 DEGs between WT and AS, with 229 up-regulated genes (56%) and 180 down-regulated genes (44%). There were 887 DEGs between WT and OE. In comparison of WT and OE, there were 444 up-regulated genes, accounting for 50.1%, and 443 down-regulated genes, accounting for 49.9% (Table 4). Each group of DEGs was drawn into a Venn diagram (Fig. 4), the number of different genes shared between the two comparison groups was 76, and these DEGs and common genes were used as candidate genes for the exploration of related genes in subsequent experiments.

**Table 4:** Statistical table of number of different expression genes.

DEG set	DEG number	Up-regulated	down-regulated
WT vs AS	409	229	180
WT vs OE	887	444	443

### Transcriptome gene expression validation

**Figure 5:**
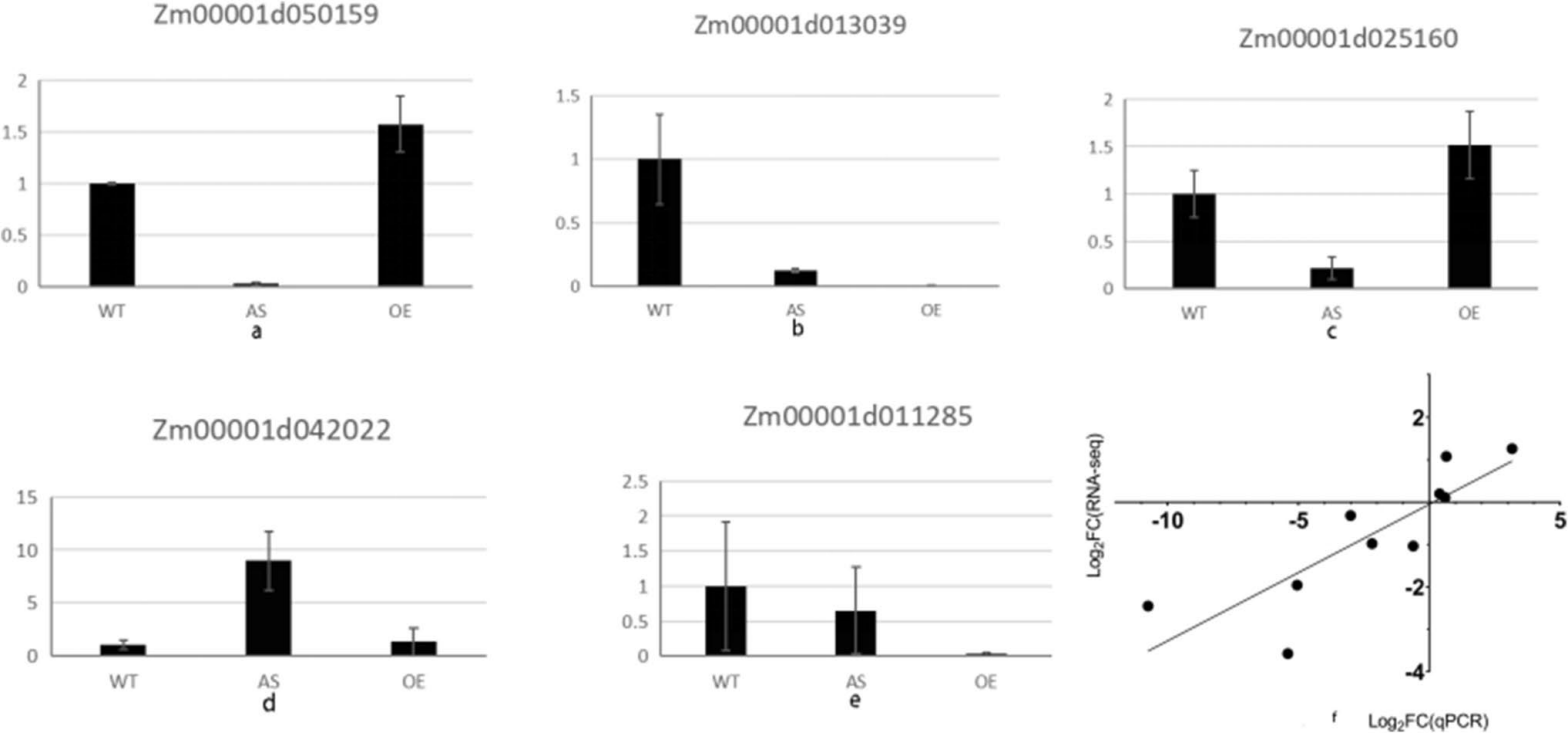
Correlation between RNA-seq and qRT-PCR gene expression levels.

Fluorescence quantitative PCR was used to analyze the gene expression profile obtained by transcriptome sequencing, and 5 genes were randomly selected to verify the transcriptome sequencing results. In this study, r^2^ > 0.8, the relative expression levels of candidate genes were highly correlated with transcriptome sequencing results, and the sequencing results were reliable (Fig. 5).

### GO classification of DEGs

**Figure 6:**
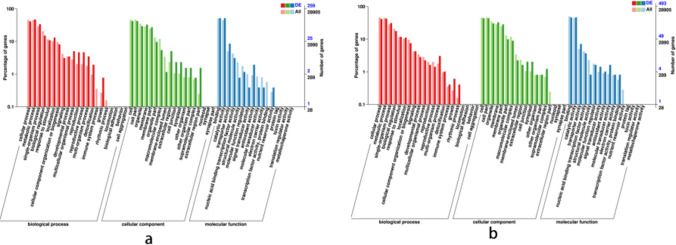
GO annotation classification statistical map of different expression genes. (**a**) Description of what is WT vs AS GO classification; (**b**) description of what is WT vs OE GO classification.

GO annotation was performed for DEGs. The biological processes (BP) were enriched in 17 GO terms. Cellular component (CC) is enriched to 15 GO terms. The molecular function (MF) was enriched to 12 GO terms. GO terms significantly enriched in WT vs AS in BP mainly include carbohydrate metabolism, lipid metabolism and tryptophan biosynthesis, etc. GO terms significantly enriched in WT vs AS in BP mainly include oxidoreductase activity, DNA binding and iron ion binding, etc. GO items significantly enriched in WT vs OE in BP mainly include RNA modification and pollen recognition, etc. while GO term significantly enriched in MF mainly includes heat shock factor binding protein and hydrolase activity, etc. (Fig. 6).

### KEGG annotation of DEGs

**Figure 7:**
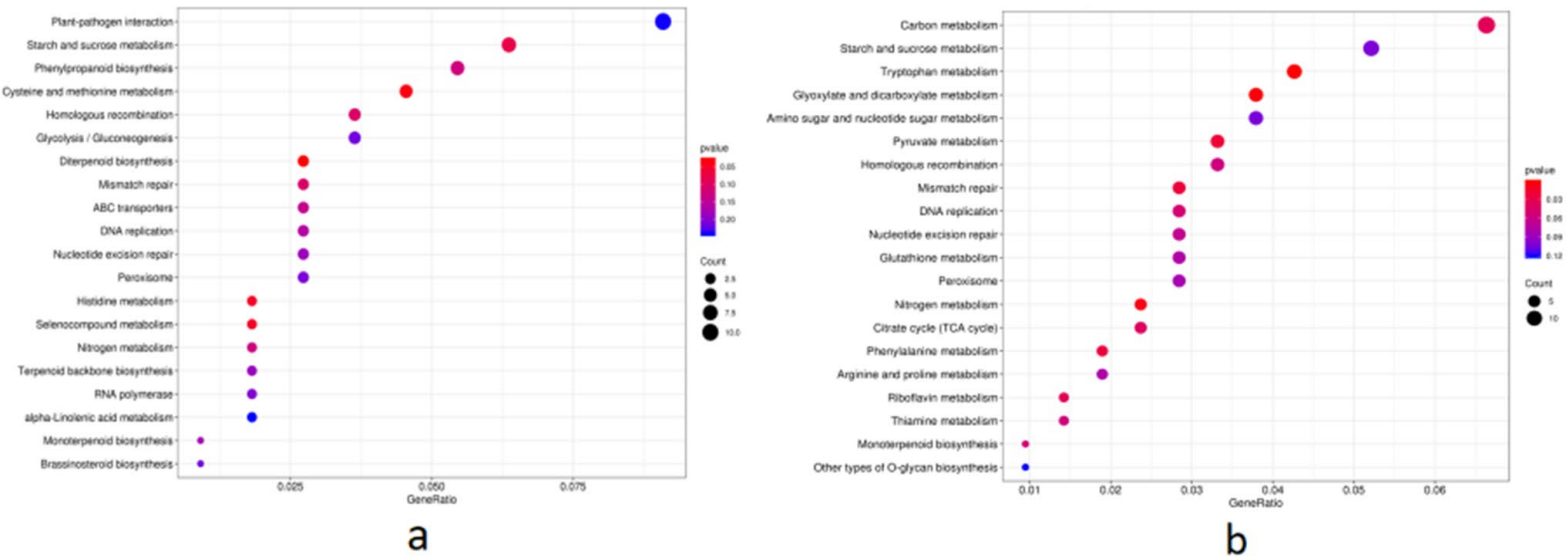
KEGG enrichment of different expression genes. (**a**) WT vs AS KEGG pathway enrichment; (**b**) WT vs OE KEGG pathway enrichment.

Through Pathway enrichment analysis of all DEGs, in the top 20 pathways with the lowest significant Q value, WT vs AS were significantly enriched in up-regulated ex-pression of DEGs in starch and sucrose metabolism, phenylpropion biosynthesis, cysteine and methionine metabolism, carbon metabolism, amino acid biosynthesis and other pathways, significantly enriched in photosynthetic antenna protein, phenylpropion biosynthesis and other pathways of down-regulated DEGs. WT vs OE was significantly enriched in up-regulated DEGs in starch and sucrose metabolism, MAPK signaling pathway, amino acid biosynthesis and other pathways, and significantly enriched in photosynthesis, porphyrin and chlorophyll metabolism and other pathways of down-regulated DEGs (Fig. 7).

## Discussion

The growth and development of plants have high requirements on environmental temperature. As sessile organisms, plants have evolved various mechanisms to sense environmental temperature and adapt to natural^28,29^. Histone methylation also plays an important role in this^30–32^. Corn is native to South America^33^, is a warm and light-loving crop, it has high temperature requirements. Its suitable growth temperature is 22–30 °C, and the seeds germinate at 28–35 °C. Under low temperature stress on different varieties of maize, the bud stage and growth stage of maize will be significantly inhibited^34,35^. Plants growing under normal conditions, the production and clearance of reactive oxygen species in cells are in a dynamic balance under the action of the protective enzyme system^36^. Under low temperature stress, MDA content will gradually accumulate with the decrease of temperature and the extension of stress time, and the protective enzyme system will undergo a series of changes^37^. The results have shown that low temperature stress has inhibitory effects on maize seed germination and seedling growth, plant height reduction, chlorophyll content and photosynthesis de-creased, physiological and biochemical indicators increased^38–41^. In the early stage of low temperature stress, it increased rapidly, and in the early stage of recovery, it decreased rapidly, with the prolongation of low temperature stress time, increasing SOD activity in maize roots, indicated that the scavenging ability of reactive oxygen species in maize was enhanced^34,42–44^. In this study, there was no significant difference in relative germination rate among the three materials under low temperature stress. Under low temperature stress, MDA content, SOD activity and POD activity were higher than those of control. Under low temperature stress, AS and OE had significant differences in MDA, but no significant differences in SOD and POD. This indicates that AS and OE are less sensitive to low temperature stress than WT, which may be caused by histone methylation modification.

Low temperature is one of the common abiotic stresses influences on plant growth, development and yield^45–47^. At the late stage of corn powder, the occurrence of low temperature will induce the generation of free radicals in plants, lead to biofilm phase transformation and change the degree of green retention of leaves^48,49^. In the later stage of pollination, higher chlorophyll content in leaves can maintain vigorous through photosynthesis, which is particularly important for improving crop quality and yield^50,51^. Expression of regulatory units of chloroplast encoding genes decreased during leaf life^52^. NSN1 (Arabidopsis nuclide-like protein 1) is involved in plant growth and development by maintaining proper cell cycle progression^53^. Short-term low temperature stress will cause physiological damage to cotton at the four- to five-leaf stage. After the low temperature stress is stopped, it can return to normal levels within 2–4 days^54^. In this study, compared with WT, the plant height of AS was significantly increased and the days of powder dispersal were significantly delayed. Compared with WT, the plant height of OE was significantly lower, and the days of OE powder were earlier. There were significant differences in chlorophyll A and total chlorophyll content between AS and WT, and significant differences in chlorophyll A, chlorophyll B and total chlorophyll content between OE and WT. In order to verify whether this phenomenon is related to histone methylation modification, the leaves at ear position of WT, AS and OE maize were selected for transcriptome sequencing 30 days after pollination. Between WT and the AS 409 DEGs, 887 DEGs between WT and OE. Through enrichment analysis, combining the GO functional annotations and KEGG corn leaf in the late pollination and growth regulation related genes are more focused on the photosynthesis, metabolism, the degradation of chlorophyll, amino acids, biological synthesis pathway.

While WT, AS genes enriched in carbon metabolism, nitrogen metabolism, starch and sucrose metabolism and phenylpropanoid biosynthesis were up-regulated, while OE genes enriched in plant hormone signal transduction pathway, photosynthesis, photosynthetic antenna protein, porphyrin and chlorophyll metabolism were up-regulated. Compared with WT, AS has a lighter degree of leaf greening in the late field, which contributes to the vigorous photosynthesis of AS in the late field, while OE has a more serious degree of leaf greening in the late field, which leads to the decline of photosynthesis. This is consistent with the previous observation of phenotype and de-termination of chlorophyll content of maize leaves at ear position. Zhao et al.'s study has shown that enzymes involved in chlorophyll degradation are significantly upregulation at the later stage of plant growth and development, and chlorophyll degradation is enhanced^55^. In Chai M's study, genes involved in photosynthesis-related and stress responses as well as the cellular components of chloroplast, PSI and photosynthetic membrane of the premature aging mutant ZmELS5 were down-regulated, consistent with chlorophyll decomposition and reduced photosynthesis^56^. The results of this study provided a theoretical basis for analyzing the growth and development of maize leaves from the perspective of histone methylation modification.

## Materials and methods

### Plant material

The seeds of the maize inbred line B73 were preserved in the maize seed resource library of Jilin Agricultural University (JLAU) in Changchun, China. Wild-type control maize inbred lines B73 (WT), SDG102 overexpressed lines OE (OE3) and SDG102 silencing lines AS (AS1) were used in this study (Fig. S1). The transgenic materials were obtained by our predecessors^57^, SDG102 silenced lines were obtained by RNAi interference, and SDG102 overexpressing plants were obtained by pollen tube channel method.

### Experimental treatment

Germination stage: 100 seeds of the same size and full were selected, disinfected with 1% sodium hypochlorite solution, and placed in a wet petri dish covered with two layers of filter paper. Two treatments were set up: 4 ℃ low temperature stress, 25 ℃ normal temperature control, three biological replicates, germination under dark conditions. The germination rate of each material was recorded.

Seedling stage: when the plant grows to the single-leaf stage, move to 4 ℃, the control is 25 ℃, three biological replicates. Physiological and biochemical indexes were determined.

Field stage: each material plant 4 rows, 15 plants per row, plant spacing 20 cm, row spacing 65 cm, field management measures are the same as those for other conventional fields.

### Determination of physiological indexes and chlorophyll content

Low temperature treatment of seed germination, seedling leaves and determination of physiological indexes. Malondialdehyde (MDA) content determination: thiobarbituric acid method^58^; the activity of superoxide dismutase (SOD) determination: nitroblue tetrazole method^59^; the activity of peroxidase (POD) determination: Guaiacol method^60^.

According to the ethanol-acetone mixture immersion method^61^, taking the fresh panicle leaf pieces of the three materials 30 days after field pollination were put into a plug scale test tube, ethanol-acetone mixture (1:1) was added, immerse the leaves completely in the liquid and cover. Place in a 30–40 ℃ thermostat, gently shake several times during the soaking process. When the leaves are completely white, determine the chlorophyll content.

### Sequencing sample selection, total RNA extraction and transcriptome sequencing

In the case of maize powder, we selected the plants with good and consistent growth status, there are used for sample sending and testing. The day of pollination was set as 0 days, and the sampling time was set as 30 days after pollination. The leaves in the middle of panicle position of each material were selected and stored in liquid nitrogen for quick freezing. Three biological replicates were performed. Total RNA was extracted from leaves using OMEGA RNA kit, and the samples were sent to Beijing Biomarker Biotechnology Co., LTD for transcriptome sequencing.

### Transcriptome data processing, screening and validation

Clean Data were filtered by Illumina high-throughput sequencing platform using HISAT2 software and the RNA-Seq clean reads were aligned to the maize genome (B73 _RefGen_v4, http://plants.ensembl.org/Zea_mays/Info/Index). The amount of gene expression detected was measured by FPKM value. DESeq2 software was used for differentially expressed analysis between sample groups. The read count levels were normalized to the reads per kilobase per million reads. DEG were defined with an ab-solute value of |log2 fold change |≥ 2 and FDR < 0.05. Through GO annotation and KEGG pathway enrichment analysis, 5 DEGs were randomly screened for qRT-PCR verification (Table 5). We used PerfectStartTM Green qPCR SuperMix kit from Beijing TransGen Biotechnology Co., LTD and SYBR dye method was used to detect by fluorescence quantitative PCR.

**Table 5:** Real-time quantitative primers for different expression genes.

Gene ID	Primer sequences
*Zm00001d050159*	F: CCCTTGAGAAGATGGTGGAATACA R: GATATGTACCTGATGGAGCCCTGAT
*Zm00001d013039*	F: CGCAGCGTGGGACTCTATGTA R: GAAAGATTCAGGTGTTATGGCAACG
*Zm00001d025160*	F: TCATCTCGCAGAAGCAGAAGTCG R: AAGAGAACGCATTGAAGTAGACGCA
*Zm00001d042022*	F: AGGACCCGACGCTGAACAAG R: GTAGGAGTAGACGAACTGGTGGAAGA
*Zm00001d011285*	F: CCTGTCAACAACAACGCCTG R: CCCTTCTTCACAAATACTGGTACATC
Actin	F: CCTGAAGATCACCCTGTGCT R: GCAGTCTCCAGCTCCTGTTC

### Quantitative reverse transcription PCR (RT-qPCR) validation

RT-qPCR verification was performed on 5 randomly selected DEGs. Primer-BLAST was used for primer design. We used the reverse transcription of total RNA was perormed by TransScript One-Step gDNA Removal and cDNA Synthesis SuperMix kit from Beijing TransGen Biotechnology Co., LTD. We used PerfectStartTM Green qPCR SuperMix kit from Beijing TransGen Biotechnology Co., LTD and SYBR dye method was used to detect by fluorescence quantitative PCR, the Actin was used as the internal ref-erence primer. The relative expressions of RNAs and targets were calculated using the 2^−∆∆Ct^ method^62^.

### Ethical approval

The collection of plant material and the performance of experimental research on such plants complied with the national guidelines of China.

## Supplementary Information

Supplementary Figure S1.

## Data Availability

The total data have been deposited into the National Center for Biotechnology Information under the BioProject number PRJNA906307.
