# Supplementary figures and images for: Effects of histone methylation modification on low temperature seed germination and growth of maize
Source: Sci Rep. 2023 Mar 30;13:5196. doi: 10.1038/s41598-023-32451-5 (PMC10063631; doi:10.1038/s41598-023-32451-5)

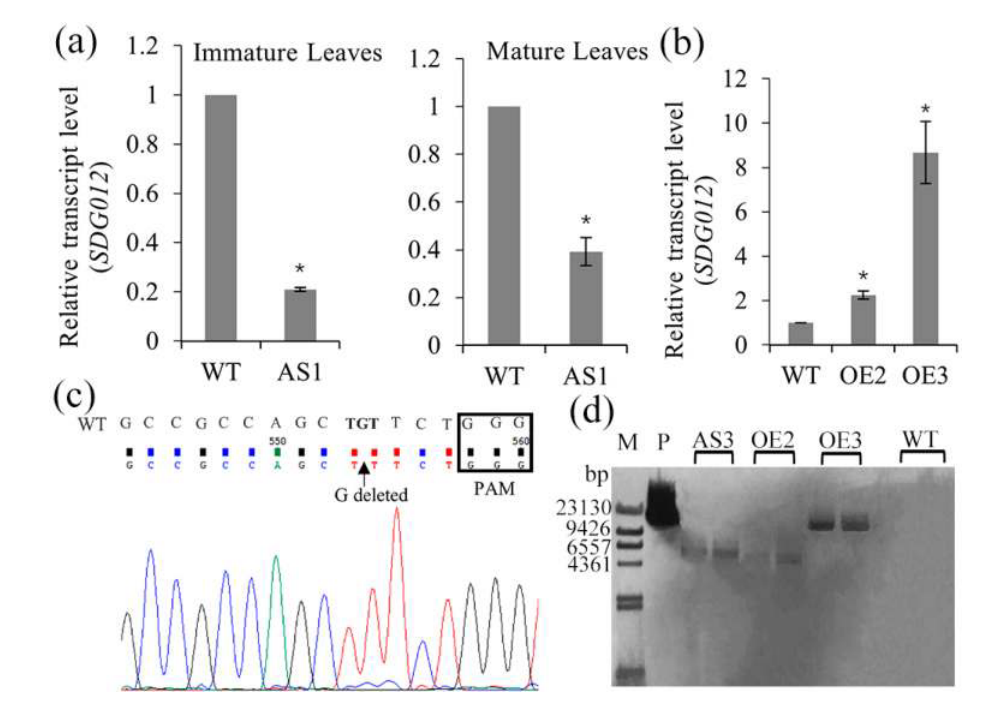

Supplement: Supplementary file 1 — Supplementary Figure S1. [file 41598_2023_32451_MOESM1_ESM.png]
